# Deciphering Cell–Cell Interactions with Integrative Single‐Cell Secretion Profiling

**DOI:** 10.1002/advs.202301018

**Published:** 2023-04-26

**Authors:** Linmei Li, Haoran Su, Yahui Ji, Fengjiao Zhu, Jiu Deng, Xue Bai, Huibing Li, Xianming Liu, Yong Luo, Bingcheng Lin, Tingjiao Liu, Yao Lu

**Affiliations:** ^1^ Department of Biotechnology Dalian Institute of Chemical Physics Chinese Academy of Sciences Dalian Liaoning 116023 China; ^2^ Key Laboratory of the Ministry of Education for Advanced Catalysis Materials Zhejiang Key Laboratory for Reactive Chemistry on Solid Surfaces Institute of Physical Chemistry Zhejiang Normal University Jinhua 321004 China; ^3^ College of Stomatology Dalian Medical University Dalian Liaoning 116044 China; ^4^ School of Pharmaceutical Science and Technology Dalian University of Technology Dalian Liaoning 116024 China; ^5^ Department of Oral Pathology Shanghai Stomatological Hospital & School of Stomatology Fudan University Tianjin Road No.2, Huangpu District Shanghai 200001 China; ^6^ Shanghai Key Laboratory of Craniomaxillofacial Development and Diseases Fudan University Tianjin Road No.2, Huangpu District Shanghai 200001 China

**Keywords:** cell–cell interactions, integrative single‐cell secretion analysis, migration, secreted factors, tumor microenvironment

## Abstract

Cell–cell interactions are the fundamental behaviors to regulate cellular activities. A comprehensive evaluation of intercellular interactions requires direct profiling of various signaling behaviors simultaneously at the single‐cell level, which remains lacking. Herein, an integrative single‐cell secretion analysis platform is presented to profile different secreted factors (four proteins, three extracellular vesicles (EV) phenotypes), spatial distances, and migration information (distances and direction) simultaneously from high‐throughput paired single cells using an antibody‐barcode microchip. Applying the platform to analyze the tumor–stromal and tumor–immune interactions with the human oral squamous cell carcinoma (OSCC) cell lines and primary OSCC cells reveals that the initial distances between cells would determine their migratory distances and direction to approach stable organization. The cell–cell in close proximity enhances protein secretions while attenuating EV secretions. Migration has a more profound correlation with protein secretions than EV secretions, in which absolute migration distance affects protein secretions significantly but not the direction. These findings highlight the significance of spatial organization in regulating cell signaling behaviors and demonstrate that the integrative single‐cell secretion profiling platform is well‐suited for a comprehensive dissection of intercellular communication and interactions, providing new avenues for understanding cell–cell interaction biology and how different signaling behaviors coordinate within the tumor microenvironment.

## Graphical abstract

An antibody barcode‐based microchip for simultaneous detection of multiplexed secreted proteins, extracellular vesicles, and migration from paired single cells is developed. Applying the microchip to analyze the tumor–stromal and tumor–immune interactions reveals the correlation between different secreted factors, physical close proximity, and migratory behaviors (distance, direction), providing new avenues for understanding cell–cell communication and interactions.

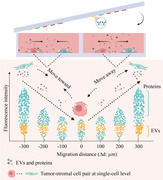

## 1 Introduction

Cells do not work alone in multicellular systems, communicating and interacting with their neighbors to constitute the population responses. Cell–cell interactions are the fundamental basis that regulates almost all physiological processes, including cell proliferation, immune responses, and tumor metastasis.^[^ ^1^, ^2^ ^]^ Recent evidences further suggest that understanding these communication and interaction behaviors could lead to new intervention strategies for diagnostic or therapeutic purposes.^[^ ^3^, ^4^, ^5^ ^]^ For example, Jayatilaka et al. reduced tumor metastasis in a mouse xenograft model by simultaneously inhibiting interleukin‐6, 8 (IL‐6/8) signaling molecules.^[^ ^6^ ^]^

Cell–cell interactions could be realized in many ways, either by cell‐to‐cell contact or through the secretion of signaling ligands to realize ligand‐receptor binding.^[^ ^1^, ^7^ ^]^ Conventional methods to decipher intercellular interactions rely on population‐based methods,^[^ ^8^ ^]^ such as ELISA (enzyme‐linked immunosorbent assay),^[^ ^9^, ^10^ ^]^ microarray,^[^ ^11^ ^]^ and RNA‐Seq (Ribonucleic acid‐sequencing),^[^ ^12^ ^]^ however, masking the cellular heterogeneity. Various single‐cell analysis technologies^[^ ^13^, ^14^ ^]^ are now being employed to decode intercellular interactions. Single‐cell RNA‐Seq data containing gene expression profiles has been used to develop algorithms to predict cellular interaction networks indirectly by matching ligand and receptor expressions.^[^ ^15^ ^]^ Since the physical proximity between cells is always lost in the single‐cell sequencing process, Giladi et al. combined cell sorting of physically interacting cells (PICs) with single‐cell RNA‐Seq to characterize intercellular communication‐specific pathways.^[^ ^12^, ^16^ ^]^ Although these studies have provided critical insights into cell interactions, transcriptional and protein‐level data usually show poor correlation, and direct measurement of signaling molecules mediating cell–cell interactions is still needed.^[^ ^17^, ^18^ ^]^ Besides, RNA‐Seq data cannot provide cell migration information, representing a critical intercellular regulator mediating a range of pathological processes, e.g., the immunological system,^[^ ^19^, ^20^ ^]^ tumor microenvironment,^[^ ^21^, ^22^ ^]^ etc. Imaging‐based detection could directly measure proteins mediating intercellular interactions and cellular spatial organization/migration.^[^ ^23^, ^24^ ^]^ However, they are usually hindered by limited signaling proteins due to spectral overlap. Multiplexed detection of secreted proteins provides an alternative way to evaluate cellular interaction networks by profiling secreted factors.^[^ ^25^, ^26^, ^27^, ^28^ ^]^ For example, Kravchenko‐Balasha et al. identified intercellular signaling through secreted proteins to induce a free‐energy gradient‐directed cell movement in a pair of glioblastoma (GBM) cancer cells.^[^ ^26^ ^]^ However, extracellular vesicles, nano‐sized particles encoding and transferring biological molecules for intercellular interaction and regulating health and diseases,^[^ ^29^, ^30^ ^]^ were not profiled simultaneously. To better understand the intercellular interactions, it is desirable to directly measure various signaling behaviors simultaneously, suggesting the great need for integrative tools for analyzing cell interaction behaviors with single‐cell resolution.

Herein, to decipher cell–cell interactions more comprehensively, we combined the antibody barcode‐based microchip and imaging to characterize different interaction behaviors between paired single cells by profiling cytokine secretion, EV secretion, physical close proximity, and migration information simultaneously. We applied the platform to analyze the tumor–stromal and tumor–immune pairwise with the human oral squamous cell carcinoma (OSCC) cell lines and primary cells, representing tumor–stromal and tumor–immune interactions within the tumor microenvironment, respectively. The unique data metric helps reveal the correlation between different secreted factors, physical close proximity, and migratory behaviors (distance, direction), providing new avenues for understanding cell–cell communication and interactions.

## 2 Results

### 2.1 The Microchip Platform to Realize Integrative Detection of Secreted Proteins, Extracellular Vesicles, and Migration from Paired Single Cells

**Figure 1:**
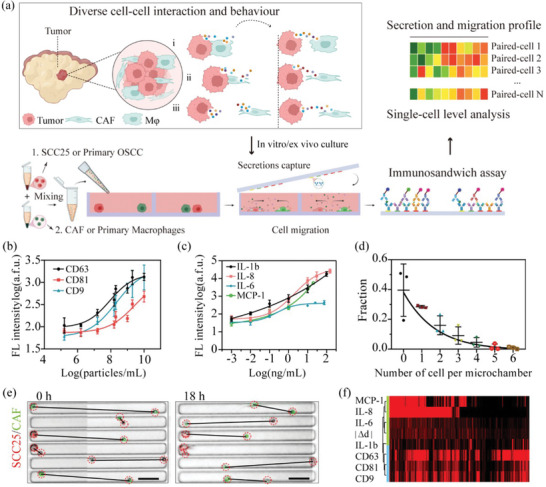
Deciphering cell–cell interactions through integrative single‐cell secretion profiling with the antibody barcode‐based microchip. a) The schematic illustrates the microchip platform and the experimental workflow to load different cell types into microchambers to form cell pairs to realize pairwise interaction for analysis, including tumor cells, CAF, and macrophages (M*φ* for macrophages). The microchambers would be imaged to record their locations to extract migration information. Their corresponding secretion profiles would be obtained via immuno‐sandwich assay on the top of high‐density antibodies barcode glass slide. b,c) Titration curves with EV standards (CD63, CD81, and CD9 phenotyped) and recombinant proteins (IL‐1b, IL‐6, IL‐8, MCP‐1). Error bar: 1SD. d) The distribution of cell numbers in 10 000+ microchambers shows that ≈20% of the microchambers would be occupied with single‐cell pairs (*n* = 3). e) In order to show the migration behaviors of paired single cells, we selected the same microchambers that trapped two cells from the large image at 0 and 18 h, respectively. Representative images show the side‐by‐side comparison of cells in the same microchambers at 0 and 18 h, showing the heterogeneous migration behaviors (red: SCC25, green: CAF, and black lines: intercellular distances). Scale bar: 100 µm. f) Representative heatmap showing the integrative secretion profiles from SCC25‐CAF cell pairs (*n* = 1013), in which each row represents a complete protein profile from a single cell/pair, and each column is a secreted factor of interest.

**Figure** **1**a shows the antibody barcode‐based microchip platform and experimental workflow to profile two types of secreted factors, spatial location, and migration information from high‐throughput paired single cells. The microchip platform comprises a high‐density microchamber array and a glass slide patterned with spatially resolved antibodies defined with microchannel guidance. Microchannel parameters, such as channel numbers, can be flexibly modified according to the number of detection targets by combining spatial and spectral encoding. In order to conveniently achieve the codetection of a full panel of secreted targets, the length of microchambers is usually two times as wide as the pitch size of full antibody barcodes. Moreover, their width and depth can also be tailored to suit the target cell's size, throughput, abundance of targets, etc. In order to meet the need for low‐abundance detection (e.g., EVs) and increase the throughput, three notable modifications were made from our previously reported devices to make the platform adapt to the cellular interaction analysis:^[^ ^28^, ^30^, ^31^ ^]^ (1) The number of long strip‐shaped polydimethylsiloxane (PDMS) microchambers was increased from 6000+ to 10 000+ to accommodate more single‐cell pairs. (2) The length of each microchamber was reduced from 1.44 to 0.62 mm to shorten the diffusion distance and increase the relative concentration of secreted factors for sensitive detection (Figure S1 (Supporting Information), dimension of each microchamber: 45 µm width by 620 µm length by 30 µm depth, the volume of each microchamber reduced from 1.7 to 0.84 nL). (3) The PDMS microchip with a high‐density paralleled microchannel array was also reduced accordingly to five microchannels (each antibody stripe 40 µm in width) to pattern antibody barcodes with high uniformity (Figure S2, Supporting Information). Three microchannels were used to coat antibodies for EV immunophenotyping, including CD9, CD81, and CD63, which are highly expressed in EV and play critical roles in regulating cell development, activation, growth, and motility.^[^ ^32^ ^]^ Meanwhile, two other microchannels were employed to immobilize antibodies for protein secretion detection, including interleukin 8 (IL‐8), interleukin 6 (IL‐6), monocyte chemoattractant protein‐1 (MCP‐1), and interleukin one beta (IL‐1b) (the antibody pairs were characterized with recombinant proteins, ensuring minimal cross‐reactivity, Figure S3, Supporting Information), closely associated with immune activation and tumor progression. These antibodies were patterned onto a poly‐l‐lysine (PLL) treated glass slide with 40 µm spatial resolution to realize 4‐plexed protein detection and 3‐plexed EV phenotyping through the combination of spatial encoding and spectral encoding (two‐color fluorescence detection). We characterized the detection sensitivity of the antibody barcode‐based platform with EV standard and recombinant proteins, respectively, which revealed that the detection limit for protein was 40 pg mL^−1^, and EV was ∼3 × 10^4^ particles µL^−1^ (Figure 1b,c). Considering the volume of each micro chamber for the assay is ∼0.84 nL, the lowest detectable number of protein molecules is 2300 molecules, and the number of EV is around 25 vesicles per microchamber, ensuring high sensitivity detection.

### 2.2 Poisson Distribution‐Based Pairing of Different Single Cells in Microchambers to Enable Cell–Cell Interactions

Oral squamous cell carcinoma (OSCC) is the most common type of oral cancer, representing about 90% of all oral malignant tumors, with an annual mortality rate of more than 50%. Diverse cell types and signaling events in the tumor microenvironment contribute to pathological progression.^[^ ^33^ ^]^ These stromal and immune cells would actively interact with tumor cells to mediate tumor progression, drug resistance, and therapeutic outcomes.^[^ ^34^ ^]^ Besides tumor cells and their neighbor cells, bacteria surround and inside tumor and immune cells,^[^ ^35^ ^]^ providing a pivotal cue for tumoral initiation, progression, and response to anticancer therapies.^[^ ^36^ ^]^ We profiled three types of paired single cells, including SCC25‐CAF (cancer‐associated fibroblasts), OSCC‐CAF, and OSCC‐macrophage, representing tumor–stromal and tumor–immune interactions, respectively. Primary OSCC and CAF cells were isolated from tissues and verified with surface biomarkers immunostaining^[^ ^37^, ^38^ ^]^ (Figure S4, Supporting Information). Primary macrophages were derived from a healthy donor. In order to simulate tumor–macrophage interaction under pathogen conditions, tumor cells and macrophages are cocultured with the RPMI 1640 medium complemented with lipopolysaccharide (LPS, a pathogen‐associated molecular pattern) (100 ng mL^−1^). To enable the pairing of single cells within microchambers, two different types of cells (stained with membrane dyes CellTracker DIO and DID, respectively) were mixed at a 1:1 ratio. In order to enhance cell adhesion and minimize nonspecific protein adsorption, the PDMS microchamber array was treated with oxygen plasma for 1 min before cell loading. Then the mixed cells were directly pipetted onto the PDMS microchamber array to realize the Poisson distribution‐based pairing of different single cells in the same microchambers. After that, the antibody barcode glass slide can be imposed onto the PDMS microchip to realize cell trapping.

The PDMS microchambers could be scanned with a Nikon microscope and merged into a large image to record the cell number and spatial information at the beginning and end of experiments. The results showed that cells would distribute randomly into each microchamber across the whole microchip, fitting Poisson distribution and ensuring we could obtain secretion information from different cell numbers. Approximately 40% of the microchambers contained zero cells, ∼28% with single cells, and ∼20% with paired single cells (*n* = 3) (Figure 1d). The paired single cells could be categorized into the same type of cells or different types, representing different intercellular interaction scenarios. The average percentage of the paired single cells with different types is 5.8% (*n* = 3), which could be increased by optimizing the concentration and the ratio of two different cells. Zero‐cell data provides the background to threshold positive secretion events. While the single‐cell data, unperturbed by other cells, provides the reference information for paired single‐cell data to evaluate the influence of intercellular interactions. The trapped cells in microchambers were incubated in a tissue culture incubator for ∼18 h to interact with each other and accumulate detectable secretions before releasing the antibody barcode glass slide from the PDMS microchip (Figure 1e). A combination of detection antibodies was applied to finish the sandwich immunoassay detection procedure, transforming captured secretomes into detectable fluorescence signals. A representative raw dataset from integrative secretions profiling of paired SCC25‐CAF single cells is shown as the heatmap (Figure 1f), demonstrating the successful implementation of the experimental workflow.

### 2.3 The Initial Spatial Distance between Paired Single Cells Determines the Migration Distance/Direction to Approach Stable Spatial Architecture

**Figure 2:**
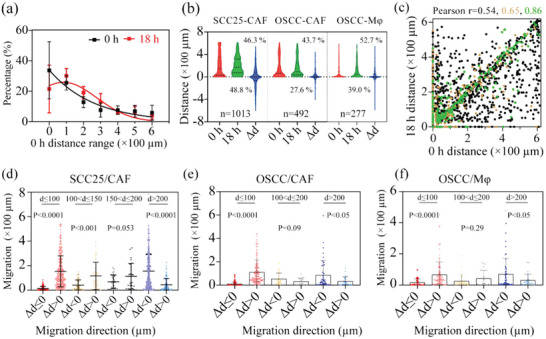
The initial distance between paired single cells determines their migration distance and direction. a) The distribution of the distances between paired single cells after loading (0 h, black square) and culture for 18 h (red square) (*n* = 3). b) Comparison of migration distances between different paired single cells. The paired single cells were classified into three groups based on the relative migration distances Δ*d* (the end distance minus start distance): Δ*d* > 0, Δ*d* < 0, and Δ*d* = 0. The percentage of their migration direction (Δ*d* > 0 versus Δ*d* < 0) was labeled in each group. Red, green, and blue colors represent the initial (0 h), culture for 18 h (18 h), and migration distance (Δ*d*), respectively. c) The correlation between paired tumor–stromal single cells distances after 18 h culture and their initial distance at 0 h. SCC25‐CAF, OSCC‐CAF, and OSCC‐macrophage were encoded as black, yellow, and green dots, respectively. Pearson *r* = −0.54 (SCC25‐CAF, *n* = 1013), 0.65 (OSCC‐CAF, *n* = 492), 0.86 (OSCC‐macrophage, *n* = 277), *P* < 0.00001. Pearson *r* values were obtained with linear regression analysis. d–f) The scatter plots compare the changes in the mean migration distances under different initial distances, which were further grouped based on migration direction. d) SCC25/CAF (*P* < 0.0001 for four subgroups. The average migration distance within each group is 13.2 µm (*n* = 117), 153.6 µm (*n* = 311), 40.7 µm (*n* = 43), 116.0 µm (*n* = 39), 69.0 µm (*n* = 38), 113.8 µm (*n* = 26), 155.8 µm (*n* = 345), 43.6 µm (*n* = 93), respectively). The fraction of the cells for four subgroups was 27.3%, 72.7% (0–100 µm), 52.4%, 47.6% (100–150 µm), 59.4%, 40.6% (150–200 µm), and 78.8%, 21.2% (>200 µm). e) OSCC/CAF (*P* < 0.0001 for 0–100 µm, *P* = 0.09 for 100–200 µm, and *P* < 0.05 for >200 µm group, respectively). The average migration distance is 9.5 µm (*n* = 213), 110.0 µm (*n* = 169), 52.1 µm (*n* = 22), 30.5 µm (*n* = 21), 85.7 µm (*n* = 42), 31.8 µm (*n* = 25), respectively). The fraction of the cells was 55.8%, 44.2% (0–100 µm), 51.2%, 48.8% (100–150 µm), 62.7%, and 37.3% (>200 µm). f) OSCC/M*φ* (*P* < 0.0001 for 0–100 µm, *P* = 0.29 for 100–200 µm, and *P* < 0.05 for 200–300 µm group, *n* = 55, 103, 25, 18, 51, 25, respectively), M*φ* for macrophage. The average migration distance is 15.8 µm (*n* = 55), 65.6 µm (*n* = 103), 25.4 µm (*n* = 25), 40.9 µm (*n* = 18), 68.8 µm (*n* = 51), 29.6 µm (*n* = 25), respectively). The fraction of the cells was 39.1%, 60.9% (0–100 µm), 59.1%, 40.9% (100–200 µm), 68.4%, and 31.6% (>200 µm). *P* values were obtained with unpaired two‐tailed *t*‐test.

We first evaluated the motility behaviors of the paired single cells in the same microchamber to understand how the spatial distance between cells influences cell movement and organization. Long strip‐shaped, transparent PDMS microchambers provide sufficient space for single cells to move around. In microfluidics, the positions of the cells would be kept consistent during the transportation of the device due to attenuation of gravity effects and increasing in surface effects in a unidirectional, steady system.^[^ ^39^, ^40^, ^41^ ^]^ We imaged and measured the distance between paired single cells at the beginning and the end of cells incubation. Their relative migration distances in the time frame can be quantified as Δ*d* using end distance minus start distance. Δ*d* can be either positive, negative, or zero, indicating the paired single cells are moving away or towards each other or not moving. **Figure** **2**a shows the initial distance between paired single cells after loading, in which we can see that the initial distance between paired single cells was distributed randomly, and 20–30% of paired single cells were distanced within 0–100 µm. Most of the paired single cells would change the distance between them after 18 h of incubation, in which 95.1% of paired SCC25‐CAF, 71.3% of paired OSCC‐CAF, and 91.7% of paired OSCC‐macrophage single cells would migrate. The average |Δ*d*| for three different paired single cells was 117.5 µm (*n* = 1013), 57.7 µm (*n* = 492), 47.5 µm (*n* = 277) for SCC25‐CAF, OSCC‐CAF, OSCC‐macrophage pairs, respectively, indicating the more substantial motility capability for paired SCC25‐CAF cells (Figure 2b).

Linear regression analysis between the initial and final distances shows that the resulting distances for paired single cells were highly dependent on their initial spatial positions (Pearson *r* = 0.54, 0.65, 0.86, *P* < 0.0001, Figure 2c and Figure S5, Supporting Information), demonstrating the significant influence of spatial location on cell movement. We binned each type of paired single cells into three subgroups based on their initial distances, and each group was further divided into two subgroups based on their migration direction: Δ*d* ≤ 0 and Δ*d* > 0 (Figure 2d–f). The results verify that the migration trend of paired single cells was dependent on their initial distances (Figure 2d). For example, if the initial distances were within 0 ≤ *d* < 100 µm, 72.7% of SCC25‐CAF paired single cells would migrate away, resulting in significantly longer migration distances (average |Δ*d*|: 153.6 µm (Δ*d* > 0) versus 13.2 µm (Δ*d* ≤ 0), *P* < 0.0001). When the initial distances were within 150–200 µm, they showed a similar motility ratio of 59.4% (Δ*d* < 0) versus 40.6% (Δ*d* > 0). Moreover, their migration distances did not show a significant difference (69.0 µm (Δ*d* < 0) versus 113.8 µm (Δ*d* > 0), *P* = 0.053). If the initial distances were more than 200 µm, 78.8% of SCC25‐CAF paired single cells migrated towards each other with longer migration distances (155.8 µm (Δ*d* < 0) versus 43.6 µm (Δ*d* > 0), *P* < 0.0001). These observations suggest that a 150–200 µm distance could be a stable cellular spatial architecture for SCC25‐CAF cells. Similar findings could be observed in paired OSCC‐CAF and OSCC‐macrophage cells distancing between 100 and 200 µm (Figure 2e,f), demonstrating that 100–200 µm could be a stable cellular distance for these cells. Altogether, these results demonstrate that the initial distances between cells encoded their motility behaviors, including distances and direction, to approach stable spatial organization.

### 2.4 Physical Close Proximity between Single Cells Enhances Protein Secretion while Attenuating EV Secretion

When two single cells are paired in an enclosed environment, they can communicate through soluble signaling ligands‐mediated paracrine signaling and physical contact.^[^ ^8^ ^]^ We asked how the paracrine signaling and physical contact between single cells would affect different secreted factors. One of the merits of the microchamber‐based single‐cell assay, compared with flow cytometry and mass cytometry, is that we could obtain the information from single cells and paired single cells from the same experiment (Figure S6, Supporting Information). Single‐cell data provides the reference information to evaluate the influence of pairwise intercellular communications. The probability of secretion from these two cells can be inferred through^[^ ^42^, ^43^ ^]^

(1) $$P\left(x|y\right)=P\left(x\right)+P\left(y\right)-P\left(x\&y\right)$$

*x* is when a single cell in a chamber secretes a particular secretome, and *y* is the probability that the other cell secretes the same secretome. Thus, the theoretical secretion frequency for each secretome from paired single cells could be calculated. Pearson correlation analysis showed that the actual secretion probabilities correlate nicely with theoretical calculations (Figure S7 (Supporting Information), Pearson *r* = 0.97, 0.97, 0.99 for SCC25‐CAF, OSCC‐CAF, OSCC‐macrophages separately, *P* < 0.01), demonstrating the synergetic communications between different single cells.

**Figure 3:**
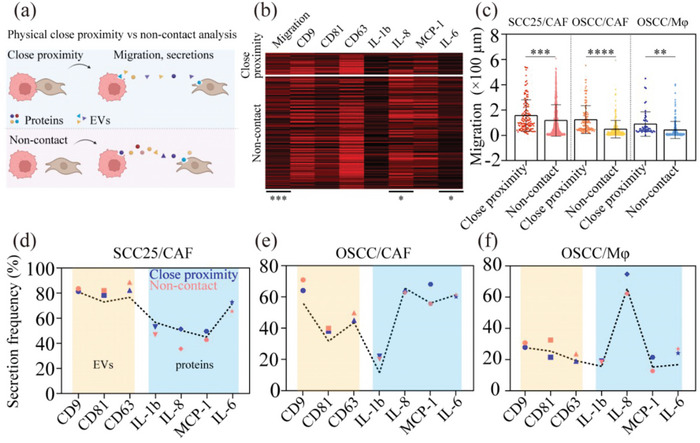
Physical close proximity between single cells plays different roles in regulating EV and protein secretions. a) Schematic shows the microchamber‐based assay to compare the measurement between contact and noncontact cells (tumor cell (red) and stromal cell (gray)). b) Heatmap comparing the raw data from paired tumor–stromal single cells with/without physical close proximity (***P < 0.001 (for migration), *P < 0.05 for IL‐8, IL‐6, respectively). c) The average migration distance of paired single cells is compared under different physical proximity conditions (close proximity/noncontact). *n* = 147, 130, 56, separately for SCC25‐CAF, OSCC‐CAF, and OSCC‐macrophage paired single cells in physical close proximity; *n* = 816, 222, 210 separately for SCC25‐CAF, OSCC‐CAF, OSCC‐macrophage paired single cells in noncontact. *P* values were also obtained with unpaired two‐tailed *t*‐test, **P < 0.01, ***P < 0.001, ****P < 0.0001, M*φ* for macrophage. d–f) Compares actual and theoretical secretion frequencies: (d) SCC25‐CAF; (e) OSCC‐CAF; (f) OSCC‐macrophage. The theoretical value of secretion frequencies for all paired cells was labeled as dashed lines. The paired single cells were classified into contact and noncontact groups based on their initial distances, and secretomes were defined as EV and protein groups (yellow for EVs and blue for proteins).

We classified paired single cells into the noncontact and close proximity groups (referring to cells with the indistinguishable boundary between them captured in microchambers under the microscope) based on their distance at 0 h, representing intercellular interactions involving paracrine signaling only and paracrine signaling plus physical close proximity (schematic: **Figure** **3**a; raw data heatmap: Figure 3b). Comparing the migration information between these two groups showed that the cells were less likely to migrate if they were initially in touch (Figure 3c). However, these cells in close proximity would migrate longer distances if they moved away, potentially due to the contact inhibition of locomotion (CIL).^[^ ^44^ ^]^ Similar migration patterns were observed in all three types of paired single cells, demonstrating the universality of cell behaviors. Besides the critical influence on cell movement, we also found that physical close proximity significantly influences secretion behaviors. In particular, we found that physical close proximity plays opposite roles in regulating EV and protein secretion (Figure 3d–f). For example, CD9, CD81, and CD63 phenotyped EV secretion frequencies decreased when two interacting cells were in close proximity (*P* < 0.001, paired *t*‐test, *n* = 9). CD63 phenotyped EV secretion would be most significantly influenced by physicalclose proximity (*P* = 0.01, paired *t*‐test, *n* = 3). However, unlike EV secretion, most of the protein secretion frequencies would increase when two interacting cells were in close proximity (*P* < 0.01, paired *t*‐test, *n* = 12), in which MCP‐1 secretion was the most significantly influenced by physical close proximity (*P* = 0.03, paired *t*‐test, *n* = 3). The differential effect of physical close proximity on secretions is potentially due to the different secretion pathways involved, in which extracellular vesicle secretion is more associated with the cell membrane that could be partially blocked when cells are in close proximity. These results demonstrate that physical close proximity between cells is critical in modulating cell movement and secretion behaviors.

### 2.5 Absolute Migration Distance Affects Protein Secretions Significantly, but not the Direction

**Figure 4:**
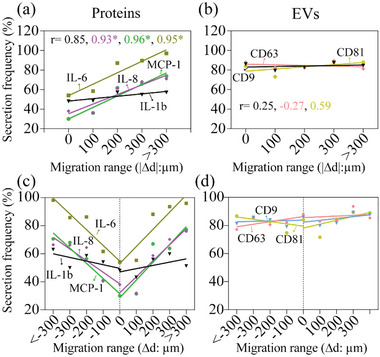
The secretions from paired single cells correlate well with migration distances but not the direction. a,b) The linear regression analysis between secretions frequency and absolute migration distances: (a) proteins, (b) EVs, *P < 0.05. c,d) The correlation analysis between secretions frequency and migration in opposite directions: (c) Proteins: Pearson *r*: −0.89*, −0.98*, −0.96*, −0.67 separately for IL‐8, MCP‐1, IL‐6, IL‐1b (Δ*d* < 0), and 0.93*, 0.92*, 0.92*, 0.56* separately for IL‐8, MCP‐1, IL‐6, IL‐1b (Δ*d* > 0), *P < 0.05; (d) EVs, Pearson *r*: 0.15, 0.68, −0.54 separately for CD9, CD63, CD81 (Δ*d* < 0) and 0.51, 0.19, 0.61 separately for CD9, CD63, CD81 (Δ*d* > 0). Pearson *r* was obtained with linear regression analysis and P values were also obtained with unpaired two‐tailed *t*‐test.

We then sought to understand the correlation between migration behaviors and secretion signatures using SCC25‐CAF‐paired single cells. We firstly analyzed the correlation between absolute migration distances and secretion frequency of proteins, which showed a strong positive correlation between them in most of the proteins (Pearson *r* = 0.93, 0.96, 0.95 for IL‐8, MCP‐1, IL‐6 separately, *P* < 0.05) (**Figure** **4**a). In comparison, the migration distances and secretion frequency of EVs were not well correlated (Pearson *r* = 0. 25, ‐0.27, 0.59 for CD9, CD63, and CD81 phenotyped EVs, respectively, *P* > 0.05), which did not change much along with varying migration distances (Figure 4b). This result showed that secreted EV had a less significant correlation with the migration distances, following previous observations that EV inhibitors did not impact cell migration.^[^ ^45^, ^46^ ^]^ Interestingly, the differential effect of migration distance on IL‐1b secretion is more like EV secretions (Pearson *r* = 0.85, *P* > 0.05) while not like proteins, whose secretion frequency did not change significantly along with varying migration distances. The potential reason is that IL‐1b is a typical protein that undergoes an unconventional secretion pathway in which vesicle trafficking is a significant pathway involved.^[^ ^47^ ^]^ Functional phenotyping of secreted factors also shows that IL‐1b is more associated with EV secretors (Figure S8, Supporting Information).

We further classified the SCC25‐CAF paired single cells into subgroups according to migration distances and direction (Δ*d* > 0, Δ*d* = 0, Δ*d* < 0), 100 µm range as one group (Figure 4c,d). Notably, most protein secretions, including IL‐6, IL‐8, and MCP‐1, were positively associated with cells' migration distances (Figure 4c). For example, IL‐8 secretion frequency from SCC25‐CAF cells would gradually increase from 30.8% to ∼75.9% when they moved away from each other from 0 to more than 300 µm. Similarly, IL‐8 secretion frequency would also increase to ∼66.4% when they move toward each other more than 300 µm. Similar correlations can be found in IL‐6 and MCP‐1 secretions (Figure S9, Supporting Information). Further analysis of the correlation between secretion frequencies and migration in opposite directions (Δ*d* < 0 versus Δ*d* > 0) revealed that the secretion of the proteins was only associated with the absolute migration distances while not with the migration direction. In comparison, secretion frequencies of vesicle trafficking pathway‐mediated secretomes, including IL‐1b, and EVs, did not change much with varying migration distances, in which similar correlation patterns in EV secretions can be found in opposite migration directions (Figure 4d; Figure S9, Supporting Information). For example, CD63^+^ EV secretion frequencies would only slightly change from 88% to 85.3% or 77.1% when they move away or towards each other from 0 to more than 300 µm. Altogether, these results demonstrate that migration distances have a varied influence on different secretion pathway‐mediated secretomes, and absolute migration distance affects protein secretions significantly but not the direction.

## 3 Discussion

This study demonstrated the antibody barcode‐based microchip to comprehensively profile various signaling behaviors between paired single cells to evaluate intercellular interactions. The platform offers some unique advantages compared to other single‐cell approaches for cell interaction analysis, including flow/mass cytometry and single‐cell RNA sequencing. In particular, simultaneously profiling different secreted factors together with spatial location and migration information would be technically challenging to obtain using other methods. High throughput paired single cells are obtained through Poisson distribution, leading to cell pairs ranging in different cellular spatial distances. Compared with controlled cell pairing methods, pairing single cells based on Poisson distribution is easy to operate, in which only pipetting and clamping are needed, without complex microchip design and complicated operating procedures. Paired single cells can move freely inside microchambers, leading to different cell communication and interaction settings, similar to in vivo conditions. Multiplexed measurements of secreted proteins and EV phenotypes can be simultaneously obtained from paired single cells for functional evaluation. Increasing evidence demonstrates the critical roles of cell‐derived EVs in mediating cell communications and regulating various biological processes, including disease progression and tumor metastasis. However, multiplexed EV secretion measurement is still a missing piece of functional characterization for paired single cells. Besides, cellular spatial organization and movement influence EV secretions have never been characterized. Our microchip platform allows us to profile the information from cell pairs for further analysis.

We applied our platform to analyze tumor–stromal and tumor–immune interactions, which revealed that the cells tend to approach stable organization through migration from the initial cellular distances. If the distance between cells is within the stable range, they will move significantly fewer distances. These observations follow previous reports that brain tumor cells would approach steady distances from each other.^[^ ^25^, ^26^, ^48^ ^]^ Our results confirmed the previous finding and extended it to paired tumor–stromal and tumor–immune cells, two types of cellular communication widely existing in the tumor microenvironment. It also demonstrated the critical roles of spatial locations in regulating cell movement and organization, which could potentially be used for intervention.^[^ ^49^ ^]^ We revealed that different types of secreted factors have a distinct correlation with cellular physical behaviors. For example, physical close proximity between cells would enhance protein secretions while attenuating EV secretions, highlighting the unique advantage of integrative profiling of different secretomes to resolve their differences. We also found that migration significantly influences IL‐8, IL‐6, and MCP‐1 protein secretions more than EV secretions and IL‐1b protein. The results suggest that the strategy to reduce cell metastasis by inhibiting signaling molecules may not work by blocking EV and L‐1b protein or other proteins that undergo an unconventional secretion pathway.

In summary, we demonstrate an integrative single‐cell secretion profiling platform well‐suited for a comprehensive dissection of pairwise intercellular interactions. The results highlight the significance of spatial organization between cells in shaping cell movement and regulating the secretion of signaling ligands. The platform readily applies to a wide range of cell types and sizes. It can also be extended to accommodate more single cells (three or four different types of single cells with distinct labeling) in the same microchambers to mimic complex cell–cell interactions in various physiological or pathological conditions better. We believe this multidimensional single‐cell interactions analysis strategy could provide new avenues to decipher cell–cell communication and interactions.

## 4 Experimental Section

### Fabrication of PDMS Microchips

The microchip molds were fabricated following standard photolithography procedures with a SU8 photoresist (Microchem, USA) to replicate PDMS microchips for antibody patterning and single‐cell capture. The silicon wafer molds were treated with TMCS (Trimethylchlorosilane, Sigma‐Aldrich) overnight to facilitate the detachment of cured PDMS (Polydimethylsiloxane) off the mold. Well‐mixed PDMS prepolymer and its curing reagent (10:1 ratio, RTV 615, Momentive) were poured onto the mold and cured at 80 °C for one hour before peeling off. The PDMS microchips were cleaned with ultrasonication in ethanol and blown dry before use.

Patterning of spatially‐resolved antibody barcodes on the glass slide. The PDMS microchip for antibody flow patterning was bonded with premium‐grade microarray glass slide evenly (poly‐l‐lysine coated, Thermo Fisher) after the inlet and outlet holes were punched out. The assembly was then baked at 80 °C for two hours to strengthen the bonding. Each antibody (Table S1, Supporting Information) was pushed through individual microchannels until completely dry with 1 psi pressured N_2_. The antibody barcode glass slide was blocked with 3% BSA (Roche, USA) for 1 h to reduce nonspecific adsorption. Then it was washed with DPBS, 50/50 DPBS/DI water, and DI water sequentially. The antibody slide was spun dry in a slide centrifuge and stored at 4 °C before use.

### Cell Isolation, Differentiation, and Culture

Human oral squamous carcinoma cell line (SCC25) from ATCC was cultured in MEM medium (Gibco; Thermo Fisher) supplemented with 10% FBS (Gibco; Invitrogen), 100 U mL^−1^ of penicillin G sodium and streptomycin, and 1% MEM Non‐Essential Amino Acid (Life Technologies). Human primary OSCC cells were obtained from the Affiliated Hospital of Dalian Medical University. Informed written consent of all participants were obtained. The Ethics Committee of Dalian Medical University approved collecting and using human samples (the assigned project number: 82 073 001 (National Natural Science Foundation of China)). OSCC cells were cultured with DMEM high glucose medium supplied with 10% FBS. FBS was ultracentrifuged at 100 000 × *g* for 4 h at 4 °C to deplete the exosome particles before use. Human primary CAF cells were isolated from human OSCC patient samples using negative selection (CD326, Miltenyi Biotec). Briefly, the tissue was minced to ≈1 mm^3^ piece and was detached with trypsin‐EDTA solution (0.25% trypsin, 0.02% EDTA) for ≈20 min at 37 °C, and then digested into flocculent tissue with collagenase I solution. After centrifugation, the supernatant was incubated with human CD326 (EpCAM) MicroBeads. Then cell sorting via magnetic separation, unlabeled cells were collected and cultured in the DMEM/F12 complete medium. The confluent cells were collected with 0.25% trypsin‐0.02% EDTA for the experiment.

Human primary monocytes were isolated from PBMC of healthy donors by using a pan monocyte isolation kit (Miltenyi Biotec) and differentiated into macrophages by culturing for 7 d with a complete RPMI medium supplied with 50 ng mL^−1^ GMCSF (R&D, USA), 20% heat‐inactive FBS. Before use, differentiated cells were harvested by 0.02% EDTA in PBS (pH = 7.2).

### Data Analysis and Statistics

After loading cells into a microchip, the whole microchip was scanned with a Nikon microscope. The cell numbers in each microchamber and the distance between cells in the same chambers were quantified with the Nikon software and listed in Excel (Microsoft). The secretion detection results were imaged with a GenePix 4300A scanner and extracted from the Genepix Pro software. The results of 7‐plexed targets were listed in Excel, corresponding to the microchamber position. The threshold for defining secretion was obtained based on the mean of the zero‐cell microchambers plus 2D. Excel and GraphPad Prism 8.0 were used for statistical analysis. Heat maps were plotted with TreeView (Eisen Laboratory).

## Conflict of Interest

The authors declare no conflict of interest.

## Author Contributions

Y.L. and T.L. designed research; H.S., L.L., Y.J., F.Z., J.D., X.B., H.L., and X.L. performed research; L.L. and H.S. analyzed data, summarized results; Y.L., B.L., T.L., and Y.L. supervised research; L.L. and Y.L. wrote the paper. All of the authors reviewed and approved the final version of the manuscript.

## Supporting information

Supporting Information

## Data Availability

The data that support the findings of this study are available from the corresponding author upon reasonable request.
