# Supplementary material for: Deciphering Cell–Cell Interactions with Integrative Single‐Cell Secretion Profiling
Source: Adv Sci (Weinh). 2023 Apr 26;10(19):2301018. doi: 10.1002/advs.202301018 (PMC10323649; doi:10.1002/advs.202301018)
Supplement: Supplementary file 1 — Supporting Information [file ADVS-10-2301018-s001.pdf]

ADVANCED SCIENCE
Open Access

Supporting Information

for *Adv. Sci.*, DOI 10.1002/advs.202301018

Deciphering Cell–Cell Interactions with Integrative Single-Cell Secretion Profiling

Linmei Li, Haoran Su, Yahui Ji, Fengjiao Zhu, Jiu Deng, Xue Bai, Huibing Li, Xianming Liu, Yong Luo, Bingcheng Lin, Tingjiao Liu and Yao Lu**

Supporting Information

Deciphering cell-cell interactions with integrative single-cell secretion profiling

Linmei Li[1,2,#], Haoran Su[1,3,#], Yahui Ji[1], Fengjiao Zhu[1], Jiu Deng[1], Xue Bai[1], Huibing Li[1], Xianming Liu[1], Yong Luo[4], Bingcheng Lin[1], Tingjiao Liu[5,6*], Yao Lu[1*]

[1]Department of Biotechnology, Dalian Institute of Chemical Physics, Chinese Academy of Sciences, Dalian, Liaoning 116023, China.

[2]Key Laboratory of the Ministry of Education for Advanced Catalysis Materials, Zhejiang Key Laboratory for Reactive Chemistry on Solid Surfaces, Institute of Physical Chemistry, Zhejiang Normal University, Jinhua 321004, China

[3]College of Stomatology, Dalian Medical University, Dalian, Liaoning 116044, China.

[4]School of Pharmaceutical Science and Technology, Dalian University of Technology, Dalian, Liaoning 116024, China.

[5]Department of Oral Pathology, Shanghai Stomatological Hospital & School of Stomatology, Fudan University, Tianjin Road No.2, Huangpu District, Shanghai 200001, China.

[6]Shanghai Key Laboratory of Craniomaxillofacial Development and Diseases, Fudan University, Tianjin Road No.2, Huangpu District, Shanghai 200001, China.

[#] These authors contributed equally to this work.

*Correspondence Authors: Tingjiao Liu: tingjiao_liu@fudan.edu.cn; Yao Lu: luyao@dicp.ac.cn.

This PDF file includes:

Figure S1- S9

Table S1

List of Supporting Information Documents:

Figure S1. The microscopy characterization of microchambers. P-3

Figure S2. The uniformity characterization of protein patterning on the poly-L-lysine-coated (PLL-coated) glass slide. P-4

Figure S3. The crosstalk test of antibody pairs. P-5

Figure S4. Characterization of Human Oral Squamous Carcinoma Cells and CAF cells with immunostaining (vimentin,pan-cytokeratin). P-6

Figure S5. Analysis of the correlation between migration distances and the initial distances between paired CAF single cells. P-7

Figure S6. Scatter plots compare the secretion results from single cells and paired single cells. P-8

Figure S7. Comparison between actual and theoretical secretion frequencies for all paired single cells. P-9

Figure S8. Scatter plot matrices show the correlation between secreted factors in paired single cells. P-10

Figure S9. The scatter plots show the relationship between migration distance and secretion frequency of SCC25-CAF paired single cells. P-11

Table S1. List of antibodies and critical reagents used. P-12

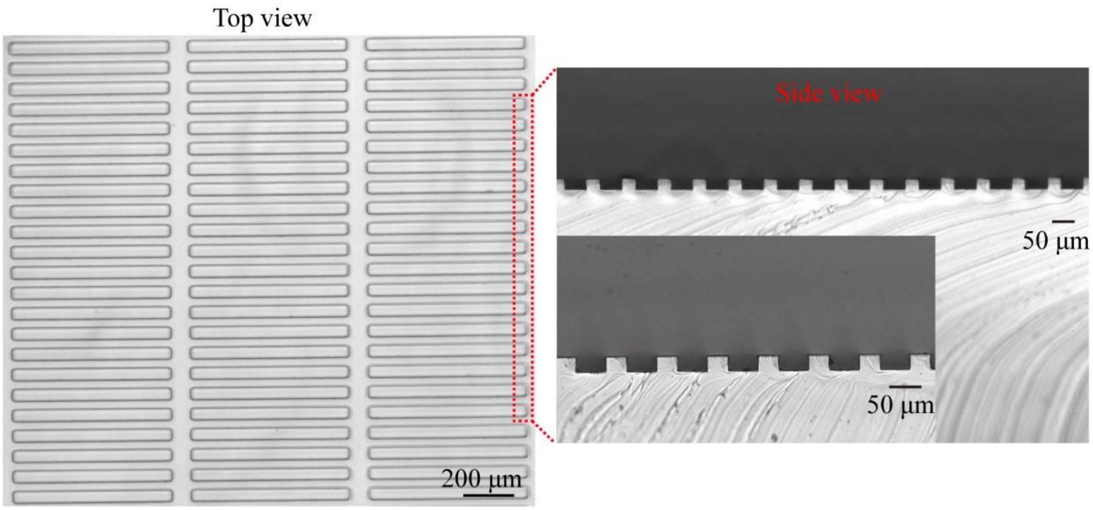

Fig. S1. The microscopy characterization of microchambers. The top image shows the addressable PDMS microchamber array for high-throughput cell culture. The enlarged side view images show the dimension of an individual microchamber: the width (~ 45 µm) and depth (~ 30 µm).

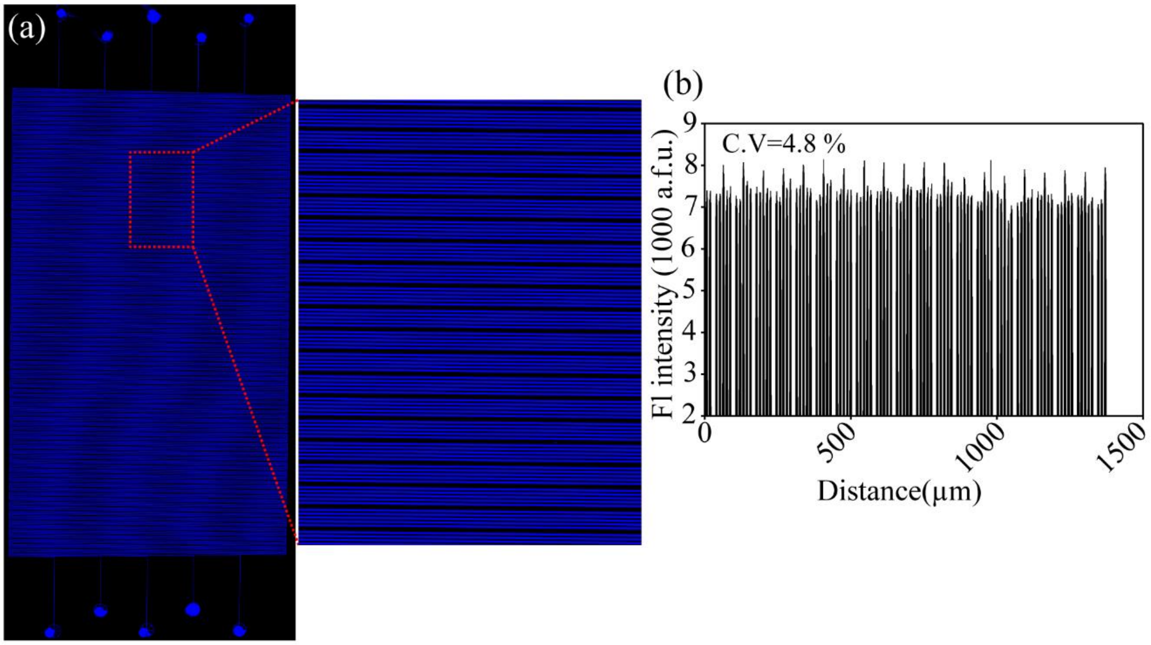

Fig. S2. **The uniformity characterization of protein patterning on the poly-L-lysine-coated (PLL-coated) glass slide.** (**a**) The scanning images show the flow patterning results of FITC-BSA across the glass slide (5-plexed), with an enlarged area marked in red. (**b**) The fluorescence intensity quantification shows the excellent uniformity of proteins/antibodies coating on the surface of the PLL-coated glass slide (C.V.= 4.8 %, n=100).

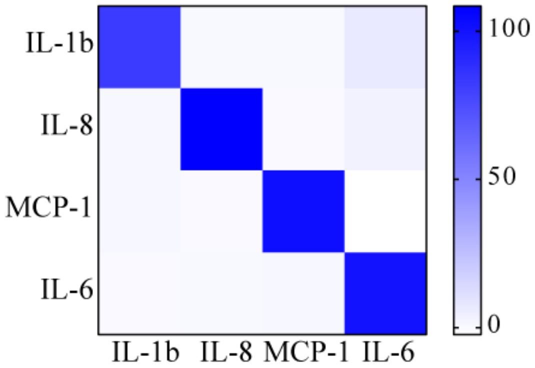

Fig. S3. The crosstalk test of antibody pairs. All the antibodies used were monoclonal to ensure minimal cross-reactivity. The capture antibodies were patterned onto the PLL-coated glass slide with parallel microchannels. Then the mixture of all standard proteins (10 ng/mL) was added and incubated, followed by detection with the combination of detection antibodies.

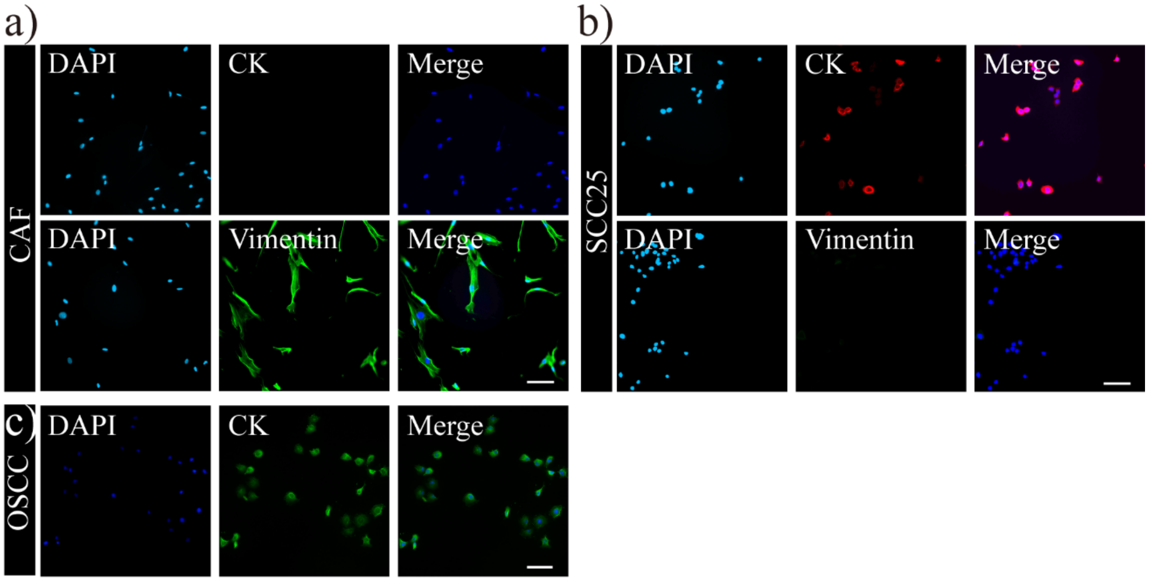

Fig. S4. Characterization of Human Oral Squamous Carcinoma Cells and CAF cells with immunostaining (vimentin,pan-cytokeratin). CAFs were identified by vimentin, and tumor cell line SCC25 and primary OSCC were identified by pan-cytokeratin based on immunofluorescent staining; (**a-c**) Images represent the immunofluorescent staining results for CAF (a), SCC25 (b), and OSCC (c), respectively. Scale bar: 100 μm.

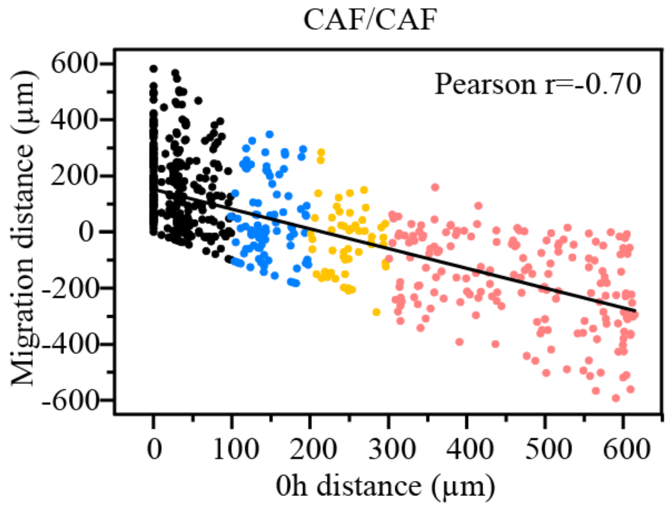

Fig. S5. Analysis of the correlation between migration and initial distances between paired CAF single cells. The regression analysis shows a good correlation relationship (r=-0.70, n=677) between migration and the initial distance of CAF-CAF cell pairs, indicating the initial distance determines the mobility capacity; Their initial distance is classified into four groups: 0-100, 100-200, 200-300, 300+ (μm), each color for one group and each dot for a pair.

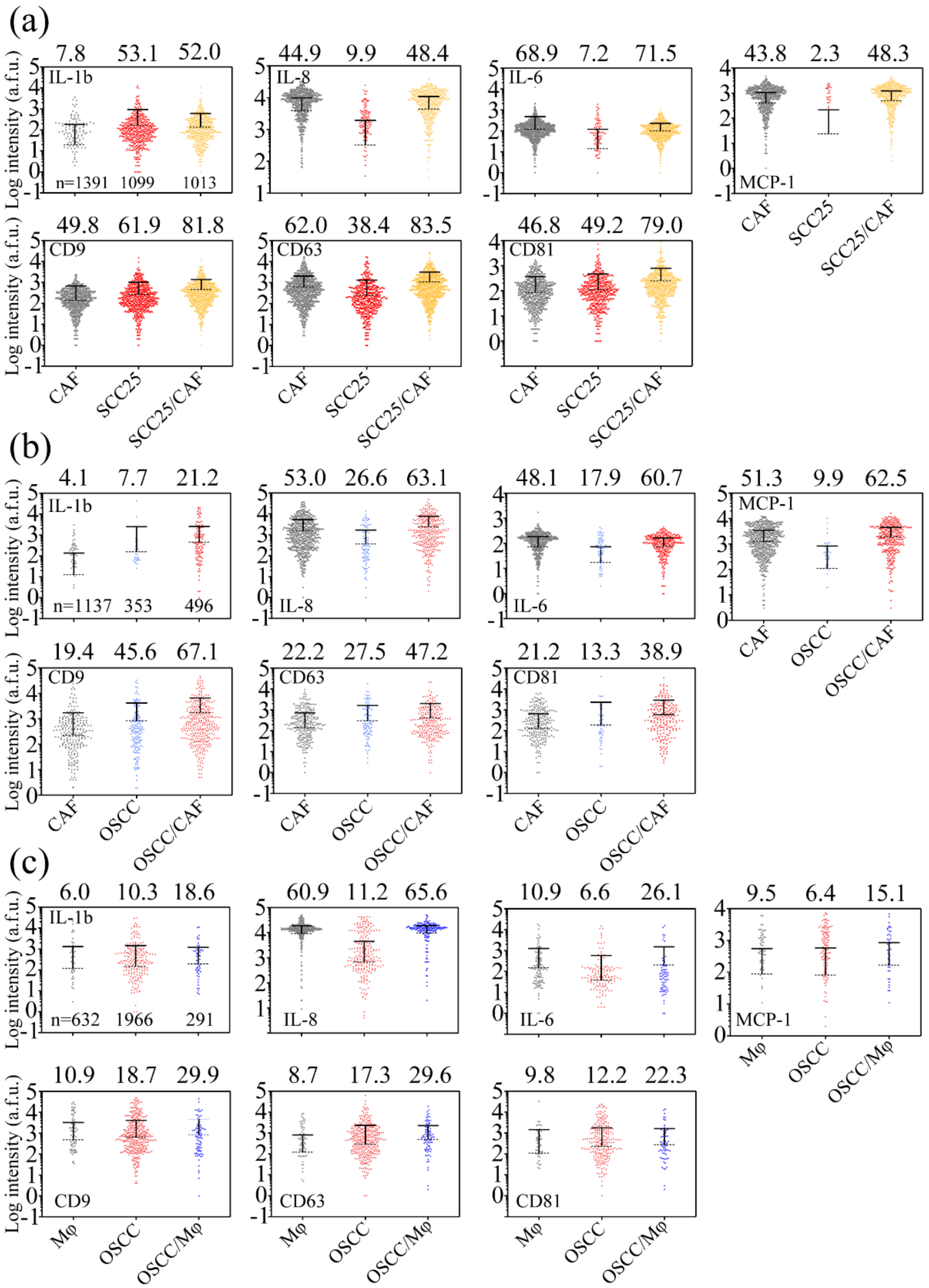

Fig. S6. Scatter plots compare the secretion results from single and paired single cells. (**a**) CAF, SCC25, and SCC25-CAF groups, (**b**) CAF, OSCC, and OSCC-CAF groups, (**c**) Macrophage, OSCC, and OSCC-macrophage groups, Mφ for macrophage. The data over the line represents the secretion frequency (%), and each dot represents a single cell. It is noted that the CAF cells in A and B were from different patients.

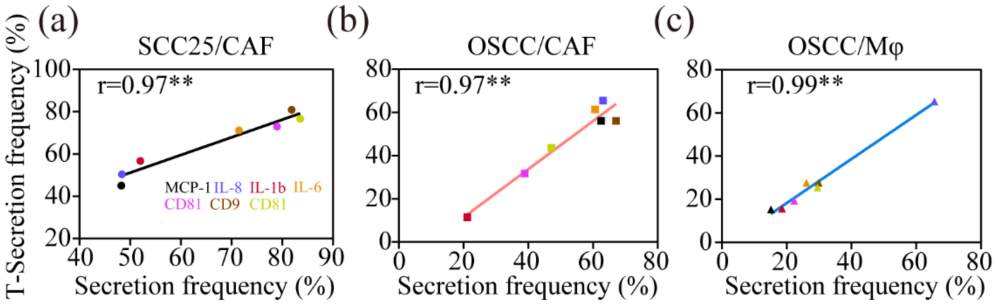

Fig. S7. Comparison between actual and theoretical secretion frequencies for all paired single cells: (**a**) SCC25-CAF, (**b**) OSCC-CAF, (**c**) OSCC-Macrophage, Mφ for macrophage, **P<0.01. The linear regression analysis shows an excellent correlation between experimental and theoretical secretion frequencies, indicating the positive synergetic interactions among tumor-stromal and tumor-immune cell pairs.

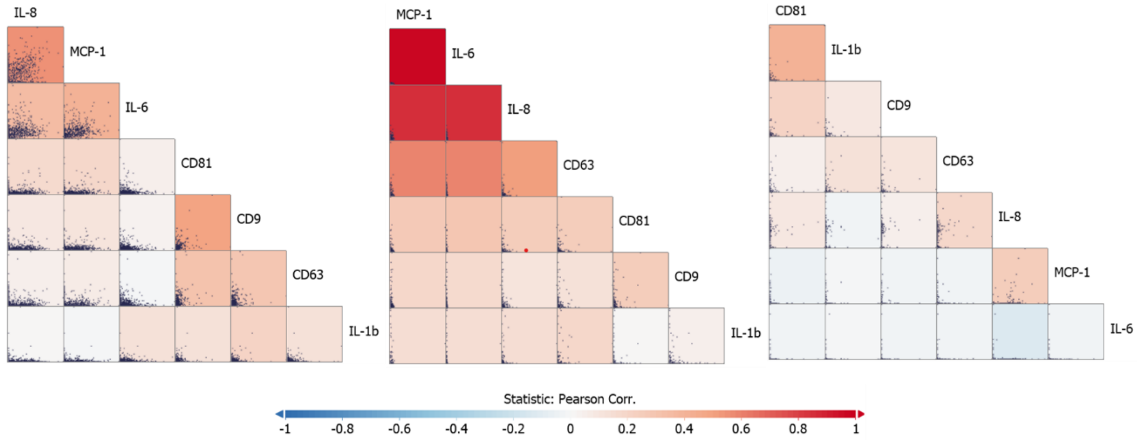

Fig. S8. Scatter plot matrices show the correlation between secreted factors in paired single cells. Each subpanel is the scatter plot showing the level of a secreted factor versus the other in all paired single cells measured. The correlation coefficient is computed through linear regression analysis. The entire matrix is color-coded by red (positive correlation) and blue (negative correlation), in which the color intensity is proportional to the coefficient value.

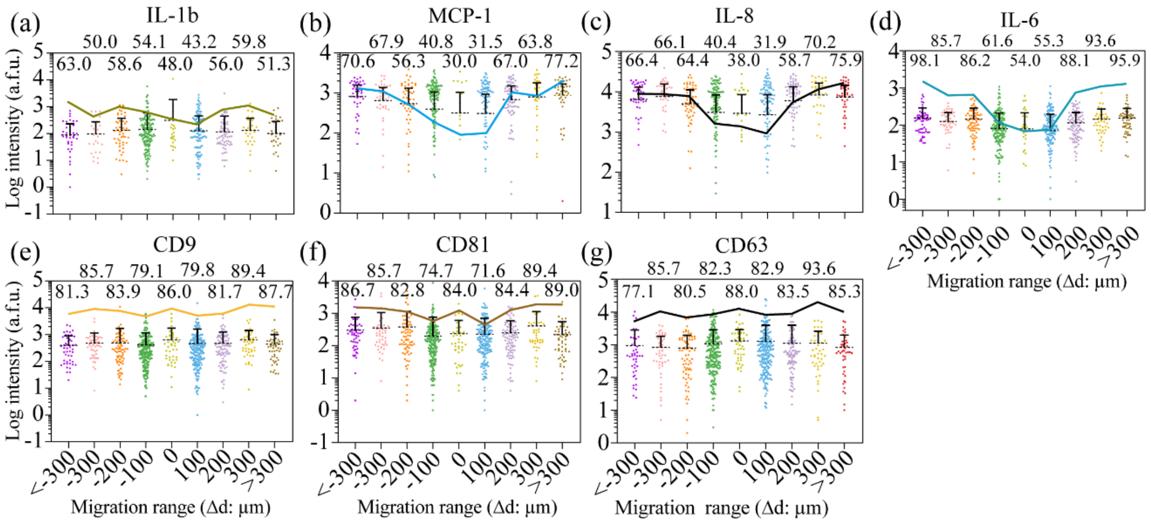

Fig. S9. The scatter plots show the relationship between migration distance and secretion frequency of SCC25-CAF paired single cells. 9 subgroups are defined based on their migration distances (100 μm range as one group) and migration direction (Δd>0, Δd=0, Δd<0). Each color represents a subgroup of migration distance, and the lines present the change of secretion frequencies listed over each subgroup.

Table S1. List of antibodies and critical reagents used.

Protein	Catalog number	Product name	Vendor
CD63	CBL553	Anti-human antibody CD63	Millipore
CD81	NB100-65805	Anti-human antibody CD81	Novus biologicals
CD9	CBL162	Anti-human antibody CD9	Millipore
EV standard	M1040	Lyophilized Exosome Standard	Biovision
IL-8	88-8086-88	Human IL-8 ELISA Ready-SET-Go!	eBioscience
IL-6	88-7106-88	Human IL-6 ELISA Ready-SET-Go!	eBioscience
IL-1β	88-7261-88	Human IL-1 beta ELISA Ready-SET-Go!	eBioscience
MCP-1	88-8337-88	Human CCL2 ELISA Ready-SET-Go!	eBioscience
Anti-human IL-6, FITC	BMS130FI	FITC anti-human IL-6 Antibody	eBioscience
Anti-human MCP-1, PE	12-7096-82	CCL2 (MCP-1) Monoclonal Antibody, PE	eBioscience
Streptavidin-APC	17-4317-82	Streptavidin APC conjugate	eBioscience
Anti-human CD63 antibody	353017-50	Biotin anti-human CD63 antibody	Biolegend
